# Stachyose triggers apoptotic like cell death in drought sensitive but not resilient plants

**DOI:** 10.1038/s41598-021-86559-7

**Published:** 2021-03-29

**Authors:** Pauline Okemo, Hao Long, Yen Cheng, Sagadevan Mundree, Brett Williams

**Affiliations:** Centre for Agriculture and the Bioeconomy, Queensland University of Technology, Brisbane, QLD Australia

**Keywords:** Genetics, Molecular biology, Plant sciences

## Abstract

Programmed cell death (PCD) is one of the most intensively researched fields in modern mammalian biology with roles in cancer, aging, diabetes and numerous neurodegenerative diseases. It is becoming increasingly clear that PCD also plays significant roles in plant defence and responses to the environment. Given their unique ability to tolerate desiccation (cells remain viable even after they’ve lost 95% of their water), resurrection plants make ideal models to study the regulation of plant PCD pathways. Previously, we showed that the Australian resurrection plant, *Tripogon loliiformis*, suppresses plant PCD, via trehalose-mediated activation of autophagy pathways, during drying. In the present study, we created a full-length *T. loliiformis* cDNA library, performed a large-scale Agrobacterium screen for improved salinity tolerance and identified Stachyose synthase (TlStach) as a potential candidate for improving stress tolerance. *Tripogon loliiformis* shoots accumulate stachyose synthase transcripts and stachyose during drying. Attempts to generate transgenic plants expressing TlStach failed and were consistent with previous reports in mammals that demonstrated stachyose-mediated induction of apoptosis. Using a combination of transcriptomics, metabolomics and cell death assays (TUNNEL and DNA laddering), we investigated whether stachyose induces apoptotic-like cell death in *T. loliiformis.* We show that stachyose triggers the formation of the hallmarks of plant apoptotic-like cell death in the desiccation sensitive *Nicotiana benthamiana* but not the resilient *T. loliiformis*. These findings suggest that *T. loliiformis* suppresses stachyose-mediated apoptotic-like cell death and provides insights on the role of sugar metabolism and plant PCD pathways. A better understanding of how resilient plants regulate sugar metabolism and PCD pathways may facilitate future targeting of plant metabolic pathways for increased stress tolerance.

## Introduction

Rainfall projections predict more frequent and severe droughts and other effects of climate change in agricultural regions worldwide. Currently, almost half of the world’s population live in areas that experience severe water scarcity at least one month a year^1^. Plants are particularly sensitive to changing environments and as such, have developed/evolved a range of tolerance strategies. Despite these adaptations, the flowering plants (angiosperms) that comprise the majority of globally cultivated crops are sensitive to adverse environmental conditions, particularly water deficit and die when their vegetative relative water content (RWC) falls from 60 to 30%. A small group of flowering plants termed resurrection plants can tolerate extreme levels of environmental stresses including excessive heat, salinity and drying (desiccation). Desiccation tolerance is the capacity of a cell or organism to equilibrate their water potential with relatively dry air (< 10% cellular relative water content (RWC) at 20 °C and a humidity of 50%)^2,3^. In nature, desiccation tolerance is widespread in orthodox seeds. Orthodox seeds tolerate drying through the reversible suspension of metabolic activity and select and controlled activation of specific plant PCD pathways. Recent studies suggest that the vegetative desiccation tolerance observed in resurrection plants is the reactivation of seed desiccation tolerance mechanisms^4–6^. Resurrection plants can ‘resurrect’ from a dry and seemingly mordant state (RWC 1%) upon watering and are found on rocky outcrops subjected to wet and dry cycles in tropical and sub-tropical climates^7,8^. An understanding of the water-use efficiency, desiccation tolerance mechanisms and how resurrection plants maintain the vitality of their cells, even when desiccated, could lead to the improvement of these traits in economically important grasses such as rice, sorghum and wheat. *Tripogon loliiformis,* a diploid, Australian native resurrection plant provides an ideal system for testing numerous hypotheses about the ecology of desiccation-tolerant plants.

Many stress tolerance strategies are either genetically coded or regulated at the molecular level, thus making resilient species not only attractive models but potential genetic sources for the dissection of stress tolerance mechanisms^3,9^. Researchers have significant knowledge of the drought responses of economically important crops; however, far less information is available on the molecular and metabolic strategies used by resurrection plants to cope with desiccation. The lack of efficient transformation systems for desiccation-tolerant plants has further hindered efforts to characterise the molecular strategies used by this small group of plants to tolerate desiccation.

Transformational mutagenesis is not possible, and although random mutagenesis using chemicals and radiation is possible, identification of the mutated gene that is responsible for the phenotype is near impossible. One method with the potential to identify molecular mechanisms driving desiccation tolerance in non-model plants is the use of gain of function mutants in model plant systems. The Full‐length cDNA Over‐eXpressing (FOX) gene hunting system is one of these methods^10^. In FOX gene hunting, libraries of cDNAs are inserted into expression cassettes and transformed into a model plant system such as Arabidopsis where they are screened directly for the trait of interest^10^.

Using a FOX screen of a *T. loliiformis* cDNA in Agrobacterium subjected to salinity stress, we identified a stress-induced stachyose synthase. Stachyose is a member of the raffinose family of oligosaccharides and a potential stress osmolyte. The raffinose family oligosaccharides (RFOs) are a group of sugars that play roles in transport, carbon storage and protection against abiotic stress^11^. Two RFOs that play vital roles in stress responses are raffinose and stachyose^12^. In most plants, raffinose accumulates in vegetative tissues during abiotic stress while stachyose accumulates predominantly during seed development/desiccation^11,13^. Some resurrection plants, however, accumulate stachyose in their vegetative tissues during drying. Why non-resurrection species mostly limit stachyose accumulation to seeds is unclear. Studies in mammalian cells linking stachyose to the induction of programmed cell death pathways (PCD), specifically, apoptosis, may provide clues^14^. We show that stachyose accumulates in *T. loliiformis* shoots during drying; however, we could not generate *N. benthamiana* plants expressing the *T. loliiformis* stachyose synthase. Exogenous application of stachyose induced typical apoptotic hallmarks, including DNA laddering and nuclear fragmentation in *N. benthamiana* plants but not in *T. loliiformis* plants. These results suggest that *T. loliiformis* encodes, and implements, measures that suppress stachyose-induced apoptotic cell death and may highlight potential gene targets for the suppression of plant PCD pathways and the production of stress-tolerant crops.

## Results

### Expression of a T. loliiformis cDNAs protect Agrobacterium from salinity

Before taking advantage of the genetic potential encoded by resurrection plants in crops for improved stress tolerance, molecular biologists must have a thorough understanding of the strategies used to confer stress tolerance. To provide further insights into the molecular mechanisms used by *T. loliiformis* to tolerate desiccation, we generated a full-length, mixed-dehydration enriched, cDNA library. The resultant library contained approximately 1.62 × 10^6^ cDNAs with an average size of 1.45 Kbp and was cloned into a plant binary vector driven by the Cauliflower mosaic virus 35S promoter. Previous studies demonstrated that the CaMV35S promoter drives the expression of plant cDNAs in Agrobacterium, thus presenting a potentially rapid system for screening stress tolerance before the generation of plants. To assess whether/which *T. loliiformis* cDNAs provide stress tolerance, we performed a preliminary salinity assay of recombinant Agrobacterium cultures harbouring the cDNA library.

**Figure 1:**
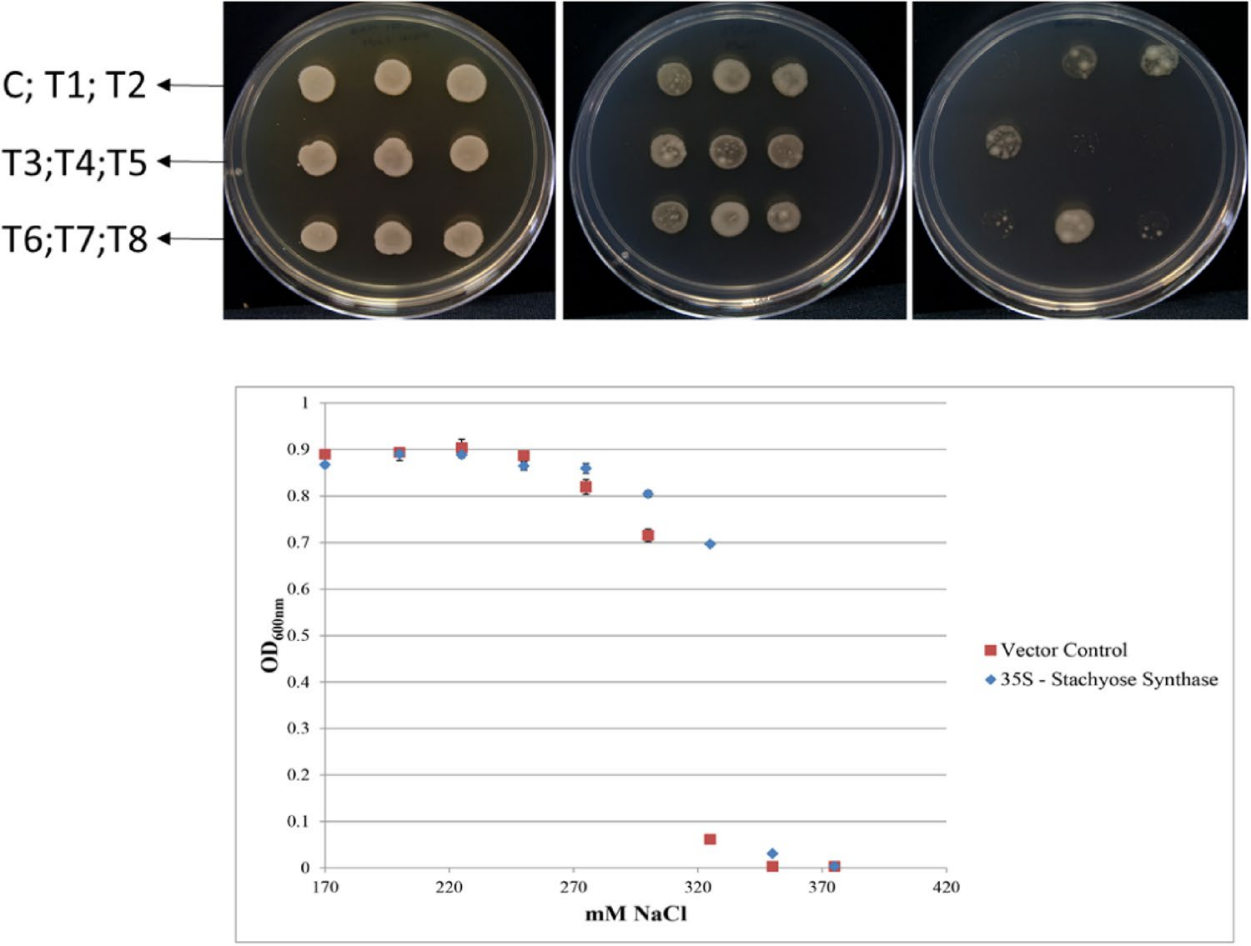
FOX screening of Stachyose Synthase genes from *Tripogon loliiformis* cDNA library in 320 mM; 345 mM and 370 Mm NaCl respectively at 48 h. Plates contain C:Control; T1–T7 *Tripogon loliiformis* stachyose synthase. Graph shows T7 (TlStach) growth in LB broth supplemented with NaCl. Optical density of bacterial growth presented as means at p < 0.05 using Minitab software version 17 (https://www.minitab.com/en-us/products/minitab/free-trial/).

Several Agrobacterium cultures displayed improved growth compared to controls harbouring an empty cloning vector. As shown in Fig. 1, one culture in particular (Tl-7), demonstrated vigorous growth on media containing 370 mM NaCl. In contrast, no growth was observed for Agrobacterium harbouring the empty vector control. Sequence analysis of the cDNA revealed an open reading frame of 758 amino acids with significant homology (81%) to *Zea mays* and *Sorghum bicolour* encoded stachyose synthases. Protein modelling displayed 100% homology to the crystal structure of an alpha-galactosidase thus, the cDNA was renamed Tl-Stach.

### Dehydrating and desiccated T. loliiformis shoots express stachyose synthase transcripts and accumulate stachyose sugar

**Figure 2:**
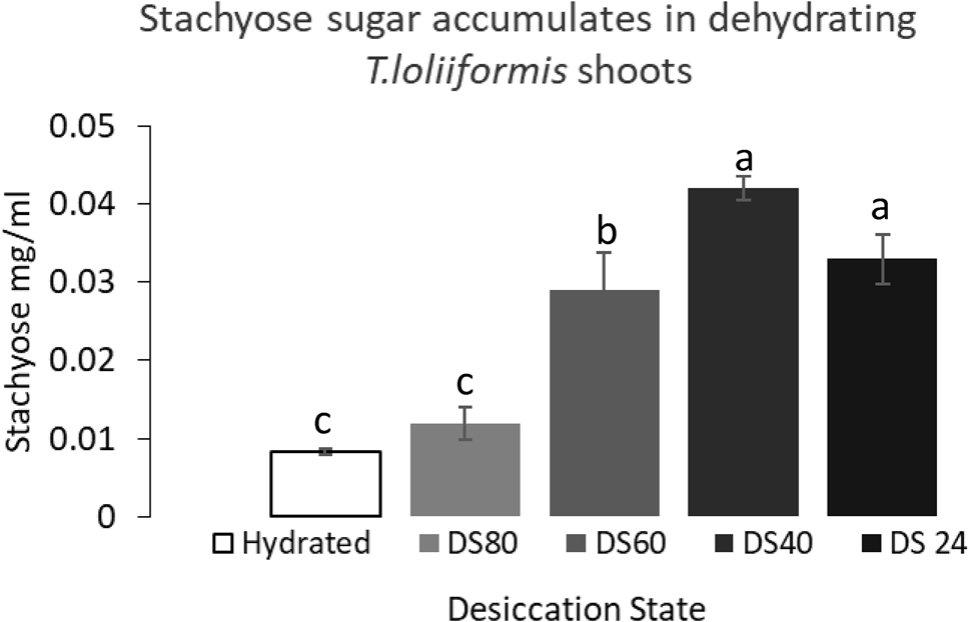
Stachyose sugar accumulation in in dehydrating *T.loliiformis* shoots. All samples measured using triplicate biological replicates. Stachyose accumulation presented as means at p < 0.05 using Minitab software version 17 (https://www.minitab.com/en-us/products/minitab/free-trial/). Samples denoted with the same letter were not significantly different from each other using p value < 0.05.

Stachyose is a tetrasaccharide member of the family of raffinose oligosaccharides that does not accumulate in the vegetative tissue of most plants but is implicated in the desiccation tolerance of seeds^15^. Conversely, at least some resurrection plants accumulate stachyose in vegetative tissues^16,17^. To determine whether stachyose accumulates in drying and desiccated *T. loliiformis* shoots, we analysed the *T. loliiformis* transcriptome and metabolome for increased accumulation of stachyose synthase, the enzyme that catalyses the synthesis of stachyose, transcripts and stachyose respectively^18,19^. The transcriptome data coupled with RT-PCR showed that dehydrating *T.loliiformis* shoots accumulate stachyose synthase transcripts (Table 1). The increased number of stachyose synthase transcripts in dehydrating shoots produced elevated stachyose levels (Fig. 2).

**Table 1:** RT-PCR and transcriptome RNA-Seq data showing fold 2 change of stachyose synthase genes expressed during dehydration in *Tripogon loliiformis* shoots.

	Hydrated	DS60	DS40	DS24
Ssynthase				
RT-PCR	1	86.698 ± 3.523	45.682 ± 4.287	5.915 ± 0.533
RNA-Seq	1	24.797 ± 7.07E−31	50.091 ± 1.17E−34	7.062 ± 1.96E−34

Analysis done in three replicates and analysed at p < 0.05 using R Studio DESeq 2 version 3.11 https://bioconductor.org/packages/release/bioc/html/DESeq2.html. Cells denoted in red and green represent increase and decrease in transcript accumulation respectively in comparison to hydrated controls.

### Stachyose may induce apoptotic-like cell death in plant cells

**Figure 3:**
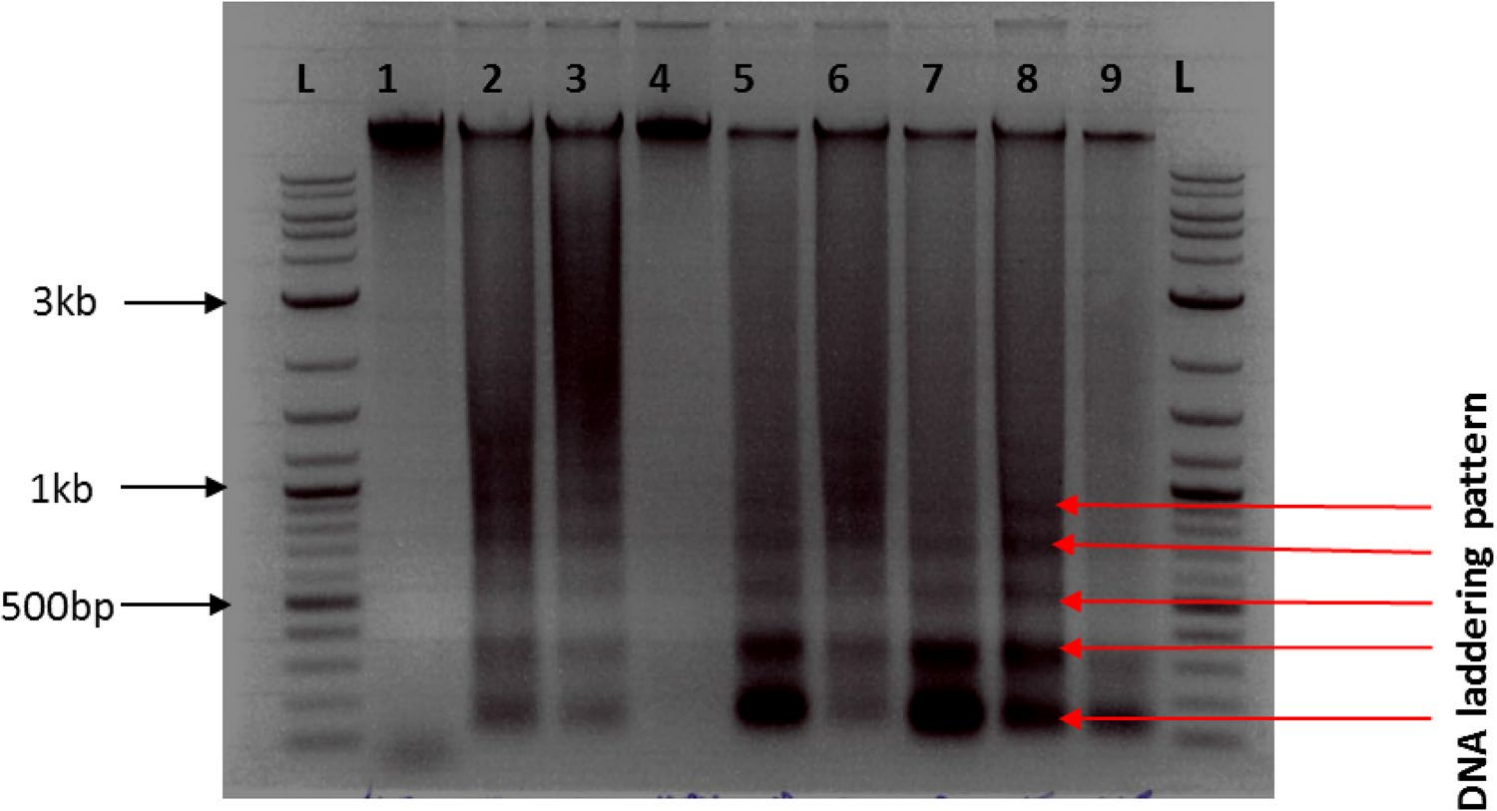
Stachyose and Trehalose induce time-dependent DNA laddering in *Nicotiana benthamiana*. DNA was extracted on *N. benthamiana* leaves treated with stachyose, trehalose and a combination of stachyose and trehalose sugars at 36 h and 48 h. 20 ng of the DNA was run on a 1% agarose gel. Potassium oxalate (KOX) treated sample was the positive control. L: 100 bp ladder; 1: untreated sample; 2/3: 20 mM KOX (36/48 h); 4/5: 5 mM Trehalose samples (36/48 h); 6/7: 3.2 mg/ml Stachyose treated samples (36/48 h); 8/9: 5 mM Trehalose + 3.2 mg/ml Stachyose (36/48 h).

**Figure 4:**
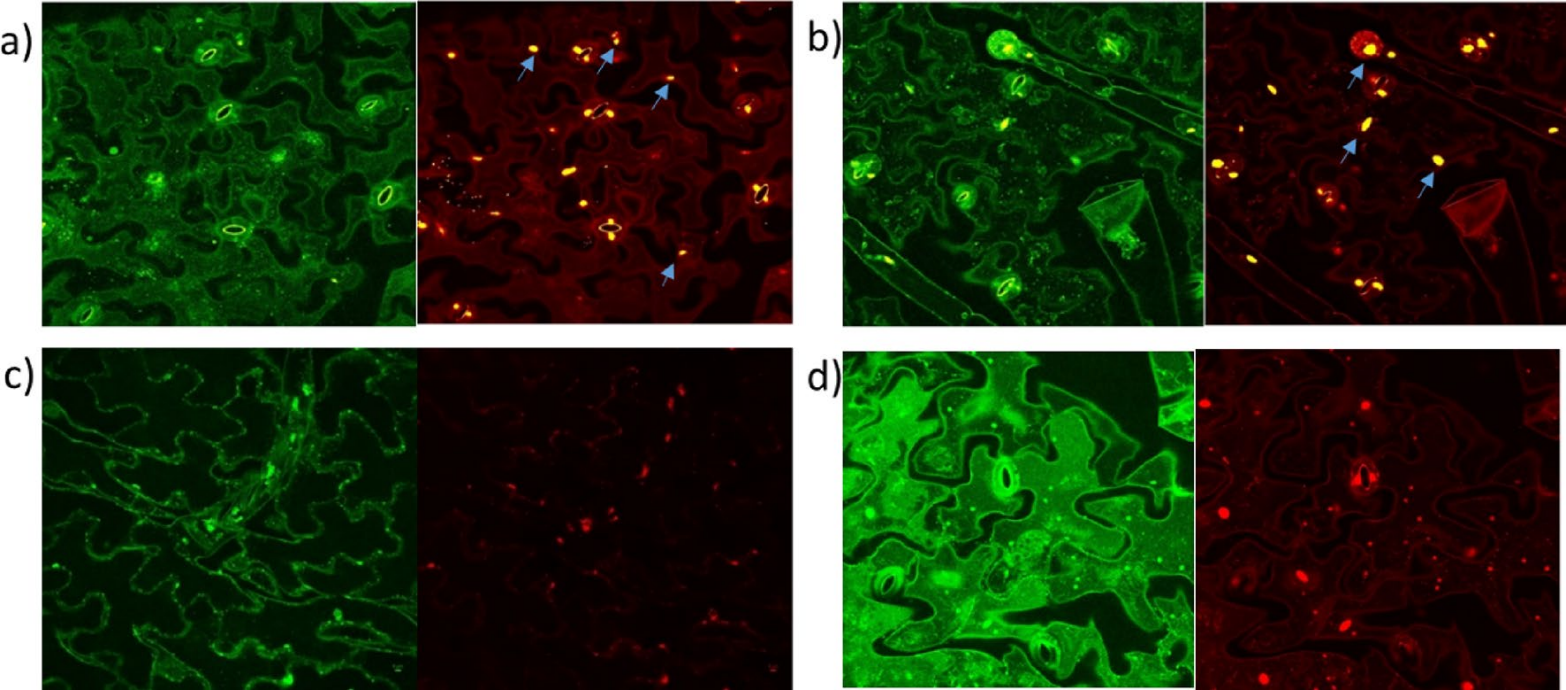
Stachyose treatment induced TUNEL positive nuclei in *N. benthamiana*. TUNEL Assays were done on sugar treated *Nicotiana benthamiana* triplicate biological replicates to detect apoptotic-like cell death. *N. benthamiana* samples treated with DNAse were used as positive controls as DNAse induces nuclei fragmentation. (**a**) 5 U/μl DNase treated samples (positive control); (**b**) 3.2 mg/ml Stachyose treated samples; (**c**) No enzyme control (Negative control); (**d**) 5 mM Trehalose treated samples (48 h). Arrows represent DNA fragmentation.

The agrobacterium screen, transcriptome and metabolome data suggest that accumulation of stachyose supports stress tolerance. To determine whether increasing stachyose content could improve stress tolerance, we attempted to generate transgenic arabidopsis and *Nicotiana benthamiana* plants expressing Tl_Stach. Despite numerous attempts and the generation of transgenic plants containing control constructs, we could not generate plants harbouring the TlStach expression vector. The inability to regenerate plants expressing a stachyose synthase raised the possibility that stachyose accumulation may be toxic. Mammalian studies have linked stachyose accumulation with the induction of apoptosis pathways in a caspase-dependant manner^14^. To determine whether we could not generate transgenic plants because of stachyose-mediated induction of plant apoptotic-like cell death pathways, we performed transient assays. *Nicotiana benthamiana* leaf tissues were infiltrated with stachyose and assessed for the presence of the apoptotic hallmarks, DNA laddering and TUNEL positive nuclei. Plants infiltrated with oxalate, an established inducer of apoptotic-like PCD, or Murashige and Skoog media (MS), were used as positive and negative controls, respectively^20^. Previously, we showed that plants display apoptotic-like features between 36 and 48 h post-stress^21,22^. As shown in Fig. 3, DNA laddering was visible in samples infiltrated with KOX and stachyose after 48 h, with more pronounced laddering observed after 36 h. TUNEL Assays detected the presence of nuclear fragmentation in stachyose treated plants (36 h/48 h), an indication of apoptotic-like cell death (Fig. 4). Despite evident laddering in trehalose treated plants at 48 h, nuclear fragmentation was absent in the trehalose treated TUNEL samples.

### Tripogon loliiformis prevents apoptosis caused by stachyose accumulation

**Figure 5:**
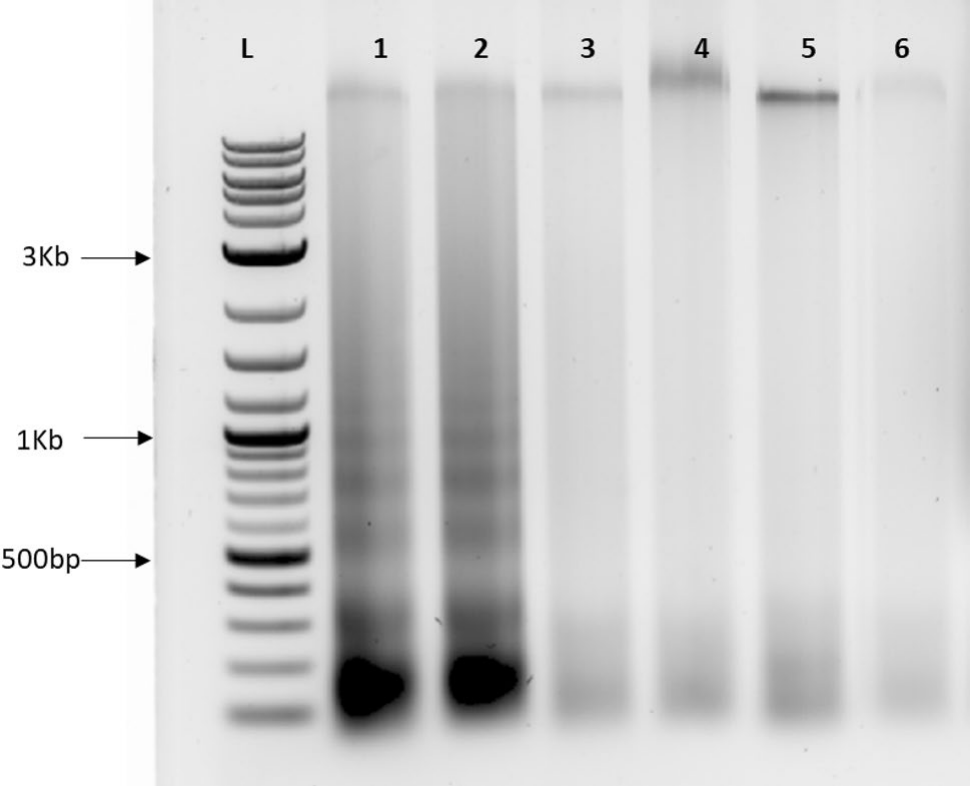
Stachyose does not induce DNA laddering in *T. loliiformis* shoots. DNA was extracted on both *N. benthamiana* and *T. loliiformis* shoots treated with stachyose at 36 h and 48 h. *T. loliiformis* shoots were also treated with 20 mM Potassium Oxalate (KOX) known to induce DNA laddering. 20 ng of the DNA was run on a 1% agarose gel. *N. benthamiana* samples treated with stachyose were positive controls. Lane 1/2: 3.2 mg/ml Stachyose treated *Nicotiana benthamiana* (36 h/48 h); Lane 3/4: 20 mM KOX treated *T. loliiformis* (36 h/48 h); Lane 5/6: 3.2 mg/ml Stachyose treated *T. loliiformis* (36 h/48 h).

Autophagy, another form of cell death is antagonistic to apoptosis. Previously, we showed that trehalose treatment induces the formation of autophagosomes, even in hydrated *T. loliiformis* leaves^18,19^. To determine whether *T. loliiformis* suppresses stachyose-mediated apoptosis, we infiltrated *T. loliiformis* leaves with stachyose and KOX and looked for the presence of DNA laddering. *N. benthamiana* samples treated with stachyose served as positive controls. No laddering was detected in *T. loliiformis* leaves infiltrated with stachyose within the same timeframe as *N. benthamiana* plants (Fig. 5). The lack of DNA laddering in *T. loliiformis* indicates that the grass has already put in place mechanisms that suppress nuclear fragmentation and DNA laddering.

## Discussion

Environmental stresses impact plants and animals in several ways. Plants, due to their sessile nature, are particularly sensitive and severely affected by environmental stresses. To combat stress, plants use a combination of strategies at the molecular, biochemical and physiological level. Accumulation of osmolytes and initiation of PCD are just a few of the physiological and metabolic changes employed by plants to protect cells against stress. Stachyose induces apoptosis in mammals. Similarly, we show that stachyose treatments induce DNA laddering and TUNEL positive nuclei, hallmark features of apoptotic-like cell death, in plants. No such features were observed following stachyose treatment of *T. loliiformis* which also accumulates stachyose in its vegetative tissue upon dehydration. We speculate that stachyose acts similarly in plant and animal cells, causing apoptotic-like cell death; however, desiccation-tolerant *T. loliiformis* utilises molecular and physiological strategies coupled with sugar metabolism to inhibit apoptosis.

Stachyose is a member of the Raffinose family Oligosaccharides (RFOs) that plays a significant role in seed desiccation^16,23^. Raffinose family oligosaccharides, including stachyose, accumulate in the late stages of soybean seed maturation and desiccation^16^. Soybean seeds induce stress tolerance through gradual drying correlating with an increase in stachyose content^24^. Arabidopsis and smaize also accumulate stachyose during seed maturation but not in vegetative tissues^24,25^. Conversely, stachyose accumulates in vegetative tissues of the resurrection plants *Craterostigma plantagineum, Xerophyta viscosa* and *Myrothamnus flabellifolia* during dehydration^26–28^. *T. loliiformis* shoots also accumulate stachyose during dehydration. Whether the accumulated stachyose sacts as an osmoprotectant in *T. loliiformis* desiccation tolerance in remains unclear.

In mammalian cells, stachyose plays a role in controlling various diseases like cancer by initiating apoptosis^29^. For instance, stachyose-induced apoptosis and expression of caspases 3 and 9 in a dose-dependent manner leading to the release of Cytochrome C (Cyt C) from the mitochondria to the cytosol. The effector caspase, caspase 3, is activated by the initiator caspase, caspase 9, via the release of Cyt C, leading to cell death^14^. A key feature in apoptotic cell death is nuclear fragmentation and DNA laddering. The laddering observed in stachyose treated *N. benthamiana* cells potentially show that despite the lack of true caspases in plant cells, parts of the pathways are conserved and stachyose acts in a similar manner to mammalian cells by inducing apoptotic-like cell death.

In yeast, trehalose acts as a chemical chaperone that prevents protein denaturation^30^. The role of trehalose in plants is less clear; however, studies suggest that it has a role in stress signalling pathways. Previously, treatment of *T. loliiformis* and *Nicotiana benthamiana* shoots with trehalose showed induction of autophagy^18,31^. Thus, suggesting that, in respect to autophagy, sensitive and tolerant plants react similarly to trehalose. Autophagy helps restore cellular homeostasis during stress sequestering cellular material for degradation and recycling of nutrients^18,32^. Due to its role in maintaining homeostasis and removing unwanted and cellular toxins, autophagy increases lifespan in mammals and delays senescence in plant cells^33–35^. By removing damaged and misfolded proteins as well as other apoptotic signals, autophagy suppresses cell death^36,37^. For instance, trehalose application induces autophagy and protects human neuroblastoma cells against Bax-induced cell death^38^. Additionally, mammalian cells displaying trehalose triggered m-TOR independent autophagy, have fewer cells showing features of apoptotic-like cell death^39^. In plants, trehalose application suppresses petal senescence and promotes the accumulation of autophagosomes in *T. loliiformis*^18,40^.

When faced with prolonged stress, some sensitive plants like *N. benthamiana* respond by accelerating their life cycle and prematurely promoting senescence. The funneling of resources in reproduction, however, renders these plants susceptible to pro-apoptotic signals and cell death. In contrast, *T. loliiformis* sutilises autophagy via regulation of the snRK1 pathway to cause an immediate shift from anabolism to catabolism. This shift is crucial and helps prevent kernel abortion and increase smaize yields^41^. *T. loliiformis* cells treated with stachyose did not undergo apoptosis as shown by lack of laddering and nuclear fragmentation. The absence of laddering is because *T. loliiformis* is primed for stress tolerance in the hydrated state and therefore activates signals that trigger autophagy early. Autophagy through the sremobilisation of nutrients provides alternative energy sources that *T. loliiformis* uses to combat stress. However, prolonged autophagy (overeating) causes the depletion of cellular machinery and may lead to cell death. In our work, the presence of DNA laddering at 48, but not 36 h post trehalose treatment may be indicative of such as case.

A key feature of resurrection plants is the suppression of apoptosis and senescence during desiccation^18,42^. One strategy that helps resurrection plants survive desiccation is transcriptional and metabolic priming in the hydrated state to enable rapid responses to stress. The early onset of dehydration adaptive strategies like autophagy may facilitate desiccation tolerance^19,43^. The cell cycle also plays a vital role in growth and development and is driven by energy metabolism^40,44–46^. The transition from the G2 to Mitosis phase is crucial and therefore acts as an essential stop-check by the cell for the presence of genetic deformities before duplication. In case the deformity cannot be repaired, PCD eliminates the cell from the organism^47^. Sugar signals regulate the major cell cycle components in meristematic cells for G2/M transition^48^. For instance, a reduction in cellular sucrose pushes the cells to the G2 phase rendering them susceptible to signals causing apoptotic-like cell death. Since the cells have been transitioned quickly to the G2 phase, they are at a high risk of acquiring abnormalities. In mammalian cells, cell death caused by cadmium application in the G2 phase displayed apoptotic-like cell death features potentially aided by changes to sugar metabolism^44,49^. Low energy levels affect plant and mammalian cells, forcing them to accelerate their life cycles. Contrary, high glucose levels promote senescence and nutrient recycling to provide alternative energy reserves required for growth and development^50^. Autophagy is employed to meet additional energy needs through sremobilisation of resources^51^. This was possibly the case with *T. loliiformis,* as a switch from anabolism to catabolism, aided by autophagy, provides the additional energy resources required to suppress apoptotic signals.

In summary, stachyose can function either as an osmoprotectant or as an inducer of apoptotic-like cell death in plant cells. However, *T. loliiformis* potentially sutilises autophagy to suppress apoptotic-like cell death. Induction of autophagy through the snRK1 pathway, as shown in *T. loliiformis*^18^ provides an alternative energy source required by the cells to maintain cellular and metabolic homeostasis during stress. Contrary, stachyose treated cells were unable to maintain cellular homeostasis and triggered apoptosis. TUNEL assays showed that stachyose induces apoptotic-like cell death in *N. benthamiana* plants. These results provide much-needed insight into the effect of stachyose on plant cells and the importance of energy metabolism in determining cell fate. How energy metabolism affects the plant cell can dictate whether a cell lives or dies when faced with developmental or environmental stresses. However, how *T. loliiformis* plants survive accumulation of stachyose and whether autophagy functions to induce signals stronger than apoptosis in dehydrating *T. loliiformis* plants warrants further research. Future work on the signalling pathways activated by stachyose application and their potential role in plant PCD should be reviewed. Additionally, further research is needed to ascertain the molecular mechanisms by which *T. loliiformis* suppresses plant PCD, including stachyose-induced PCD observed in sensitive plants.

## Materials and methods

### Plant materials and cultivation

*Tripogon loliiformis* (F.Muell.) C.E.Hubb. plants (Queensland Herbarium voucher accession number: Williams 01) were identified and collected under licence from Charleville (GPS: –26.42686 S, 146.25002E), Queensland, Australia, by Stephen Peck (QLD Parks and Wildlife) and Dr Brett Williams. The plants were germinated from seeds collected from a single plant and grown in a chamber at 27 °C and 16 h photoperiod. Twenty-one, 65 mm pots, each containing several tufts of *T. loliiformis,* were grown for two months. Before dehydration, all plants were watered to saturation. Hydrated controls were randomly collected in triplicate, 1-day post-watering. The remaining plants were subjected to water deficit by withholding water until they reached an air-dry state and a relative water content (RWC) less than 10%. Triplicate samples were collected at 80, 60, 40, and < 10% RWC throughout the dehydration. The plants were watered and rehydrated samples were collected after 48 h. The RWC was determined on *T. loliiformis* shoots and roots and was calculated according to Barrs and Weatherley (1962) using the formula (RWC (%) = ((Fresh weight − Dry weight)/(Turgid weight − Dry weight)) × 100). All the shoot and root samples were snap-frozen in liquid nitrogen and stored at − 80 °C until analysed^19^. For exogenouse application so stachyose, *T. loliiformis* shoots were infiltrated with 1.6 mg/ml and 3.2 mg/ml stachyose before subjection to analysis for DNA laddering.

Non-transgenic *Nicotiana benthamiana* seeds, collected from a single plant, were germinated in soil and grown in a chamber at 27 °C under a 16/8-h light/dark cycles at 100 μE/m^2^/s. Leaves were infiltrated with 20 mM Potassium oxalate, 5 mM trehalose, 1.6 and 3.2 mg/ml stachyose and a combination of 5 mM trehalose and 3.2 mg/ml stachyose. Samples were collected 36hrs and 48 h post-infiltration and used for either DNA laddering or TUNEL Assays.

### Total RNA extraction and high throughput sequencing

For sequencing analysis, total RNA was isolated from the shoot tissues of triplicate; hydrated, dehydrating, dehydrated and rehydrated *T. loliiformis* plants using a modified Trizol (Invitrogen) and spin column (Qiagen) method. The Bioanalyzer (Agilent technologies) was used to verify RNA integrity and quality. For library preparation, polyadenylated RNA was enriched and chemically fragmented; cDNA was ssynthesised using an Illumina RNA-seq kit according to the manufacturer’s instructions. Sequencing of the cDNA libraries was performed at Texas A&M AgriLife Genomics and Bioinformatics service, United States using an Illumina HiSeq 2500 Sequencer (Illumina, Inc.). 100 bp single-read sequences were collected^18,19^.

### RNA-seq analysis

Quality control of the sequences was performed. Primer and barcode sequences were removed by trimming. A de novo assembled and blast annotated *T. loliiformis* transcriptome assembly served as the reference for RNA-Seq profiling of the independent cDNA libraries^18^. Over 80% of the reads from each sample were mapped. All data sets were paired and used in an in silico microarray experiment using CLC genomics workbench. Using the hydrated samples as a reference for unstressed gene expression, each data set was enriched for genes that had a fold change > 2 or < − 2. Prior to analysis, the data were subjected to quantile normalisation. The distributions of the expression values for each replicate were used to create a common target distribution. The targeted distribution was then used to calculate normalised expression values^18,19^. Fold 2 change and statisticical analysis at p < 0.05 was done using R Studio DESeq 2.

### Quantitative real-time PCR analysis

cDNA was generated from 0.8 μg of Total RNA using an oligo (dT) (100 ρmol) primer and Superscript III Reverse Transcriptase (Invitrogen). Quantitative PCR was done using a ViiA7 Real-Time PCR System and the SYBR Green PCR Master Mix kit (Applied Biosystems) according to the manufacturer’s instructions using 300 mM primer and 1/100 dilution of cDNA and standard cycling parameters. Stachyose synthase primers were designed using Primer3 bioinformatic software (MIT) and are listed in Supplementary Table 1. The data analysis was completed using ExpressionSuite Software (Life Technologies). The *Tripogon loliiformis* homologue of Arabidopsis Actin identified from the annotated transcriptome were used for quantitative normalisation. Fold changes were calculated against hydrated tissue^18,19^.

### GCMS analysis

*Tripogon loliiformis* shoots at the various dehydration points described above, were sampled and analysed for sugar content by Gas Chromatography-Mass Spectroscopy (GCMS). Approximately, 100 mg of dry tissue was analysed using a Waters Acquity UPLC coupled to a mass spectrometer. For quantitation purposes, stachyose standards were analysed alongside the samples. Chromatographic separation was performed using a Waters BEH Amide UPLC column (1.7 mm, 2.1 mm 3 150 mm; 30C). The mobile phase used a mixture of water (Fisher Optima) containing 0.1% formic acid (solvent A; Fisher) and acetonitrile (Fisher Optima) containing 0.1% formic acid (solvent B) in a linear gradient from 80% B to 50% B at a flow rate of 0.35 ml/min. For statistical significance, triplicate samples were measured^19^.

### Salinity assays of recombinant Agrobacterium harbouring the T. loliiformis cDNA library

Recombinant *Agrobacterium tumefaciens* cultures harbouring expression cassettes of individual *T. loliiformis* cDNAs were grown in Luria Bertani (LB) broth containing 50 mg/l Kanamycin. Recombinant cultures containing an empty cloning vector were used as controls. The recombinant cultures were grown on LB solid media with 170, 225, 300 and 400 mM sodium chloride and incubated at 28 °C for 48 h. Comparisons were made by observing differences in growth between the recombinant cultures grown on the salinity media, as mentioned above.

### DNA laddering

Leaf tissues of *N. benthamiana* treated with 20 mM Potassium oxalate, 5 mM trehalose, 1.6 and 3.2 mg/ml stachyose and a combination of 5 mM trehalose and 3.2 mg/ml stachyose respectively and *T. loliiformis* treated with 1.6 mg/ml and 3.2mgml of stachyose as described above were snap-frozen in liquid nitrogen and ground to a fine powder. Extraction of DNA was performed using a modified CTAB protocol^52^. For analysis of DNA laddering, equal amounts of DNA (20 µg) were treated with 25 μg/ml DNase-free RNase A (Boehringer Mannheim) and incubated for one h at 37 °C. Following RNA digestion, the DNA was separated by electrophoresis on a 1% (w/v) agarose gel, and visualised by staining with 0.5 μg/ml Gel Red (Biotium) and observation under UV light.

### TUNEL assays

*Nicotiana benthamiana* samples were infiltrated with stachyose and trehalose and harvested for TUNEL assays. As a positive control, samples were treated with 5 U/μl DNase I for 10 min at 37 °C. In situ nick end labelling of nuclear DNA fragmentation was performed at 37 °C in a humid chamber for 1 h in the dark using the fluorescein, in situ Cell Death Detection Kit (Boehringer Mannheim, Mannheim, Germany) at a dilution of 1:2 in reaction buffer. For negative controls, stachyose and trehalose treated samples were labelled in parallel as above, except for the absence of TdT enzyme. After labelling, the samples were mounted on slides and rinsed in 1X PBS and counterstained with propidium iodide (PI). Confocal microscopy was performed using a Nikon A1 microscope. Seven samples were viewed under the microscope for each treatment.

### Data analysis

Data on stachyose concentration and optical density of bacterial growth were collected and analysed using Minitab software Version 17 https://www.minitab.com/en-us/products/minitab/free-trial/. ANOVA was carried out to determine the significance at p < 0.05.

## Supplementary Information

Supplementary Table 1.
