# Supplementary material for: Stachyose triggers apoptotic like cell death in drought sensitive but not resilient plants
Source: Sci Rep. 2021 Mar 29;11:7099. doi: 10.1038/s41598-021-86559-7 (PMC8007635; doi:10.1038/s41598-021-86559-7)
Supplement: Supplementary file 1 — Supplementary Table 1. [file 41598_2021_86559_MOESM1_ESM.doc]

# Title: Stachyose triggers apoptotic like cell death in drought sensitive but not resilient plants.

**Authors:** Okemo, Pauline^1^, Long, Hao^1^, Cheng, Yen^1^, Mundree, Sagadevan^1^ and Williams, Brett^1*^

**Corresponding author**: Brett Williams ([b.williams@qut.edu.au](mailto:b.williams@qut.edu.au))

**Contact information**

**^1^** Queensland University of Technology, Centre for Agriculture and the Bioeconomy, Brisbane, QLD, Australia

Supplementary Table 1

| **Primer Name** | **Sequences** | **Target** |
| --- | --- | --- |
| TlStaF | gcgtccgtcgcgtaca | Stachyose Synthase |
| TlStaR | cgggtcgcgcgtgta |  |
